# Risk Factors, Incidence, and Outcomes Associated With Clinically Significant Airway Ischemia

**DOI:** 10.3389/ti.2024.12751

**Published:** 2024-05-10

**Authors:** Gloria Li, Zejian Liu, Marcelo Salan-Gomez, Emma Keeney, Ethan D’Silva, Babith Mankidy, Andres Leon, Aladdein Mattar, Abdusallam Elsennousi, Jennalee Coster, Anupam Kumar, Bruno Rodrigues, Meng Li, Alexis Shafii, Puneet Garcha, Gabriel Loor

**Affiliations:** ^1^ Department of Medicine, Baylor College of Medicine, Houston, TX, United States; ^2^ Department of Statistics, Rice University, Houston, TX, United States; ^3^ Department of Surgery, Baylor College of Medicine, Houston, TX, United States; ^4^ Department of Cardiothoracic Surgery, University of Pittsburgh Medical Center, Pittsburgh, PA, United States; ^5^ Department of Medicine, University of Minnesota, Minneapolis, MN, United States

**Keywords:** lung transplant, airway stenosis, airway ischemia, airway anastomosis, airway dehiscence

## Abstract

Airway complications following lung transplantation remain an important cause of morbidity and mortality. We aimed to identify the incidence, risk factors and outcomes associated with clinically significant airway ischemia (CSAI) in our center. We reviewed 217 lung transplants (386 airway anastomoses) performed at our institution between February 2016 and December 2020. Airway images were graded using the 2018 ISHLT grading guidelines modified slightly for retrospective analysis. Airways were considered to have CSAI if they developed ischemia severity >B2, stenosis >50%, and/or any degree of dehiscence within 6-months of transplant. Regression analyses were used to evaluate outcomes and risk factors for CSAI. Eighty-two patients (37.8%) met criteria for CSAI. Of these, twenty-six (32%) developed stenosis and/or dehiscence, and 17 (21%) required interventions. Patients with CSAI had lower one-year (80.5% vs. 91.9%, *p* = 0.05) and three-year (67.1% vs. 77.8%, *p* = 0.08) survival than patients without CSAI. Factors associated with CSAI included younger recipient age, recipient diabetes, single running suture technique, performance of the left anastomosis first, lower venous oxygen saturation within 48-h, and takeback for major bleeding. Our single-center analysis suggests that airway ischemia remains a major obstacle in contemporary lung transplantation. Improving the local healing milieu of the airway anastomosis could potentially mitigate this risk.

## Graphical Abstract

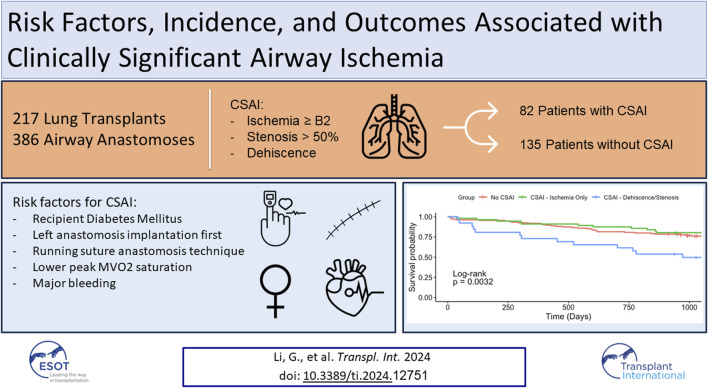

## Introduction

Lung transplant is an effective treatment for patients with irreversible lung disease, but impaired airway healing remains a constant threat affecting patient outcomes. Perfusion to the airway anastomosis relies on collateral vessels, fed from the pulmonary artery circulation in a retrograde fashion; therefore, ischemia is inevitable. While many ischemic lesions are not clinically significant, some are severe enough to warrant intervention. Severe airway ischemia leading to dehiscence and/or stenosis may require balloon dilation, stents, or operative interventions and have been associated with reduced long-term survival [1–4].

Accurate assessment of the risk of airway complications is important for clarifying the clinical sequelae and identifying preventive strategies. In the early days of lung transplant, airway complication rates were reported to exceed 50% and were associated with substantial morbidity [5, 6]. Risk factors included rejection, limitations in organ preservation, and tracheal anastomosis. Despite improvements, there has been considerable variability in the reported incidence of this complication in contemporary series. The past 15 years have seen reports of airway complications ranging from as low as 1.4% to as high as 38% [1, 3, 7–10]. This variability stems mostly from a lack of consensus around the classification of anastomotic lesions [11].

Several grading systems have been proposed to report airway complications including the TEGLA classification by Chajed et al. [12], the six category airway complications system by Santacruz and Mehta [13], and the MDS grading system by the French Language Pulmonary Society [14]. However, there are pitfalls to each method, and none has been universally adopted. To address this, a working group of the ISHLT convened in 2018 to create a consensus document to standardize airway assessments [15]. Reports demonstrating the utility and clinical integration of the updated guidelines are lacking. Such reports are needed to revisit and validate previously reported donor, technical, and postoperative risk factors while identifying potentially novel risk factors [1, 2, 7, 8, 16–20]. Studies integrating the updated guidelines could provide new benchmarks for the incidence of airway complications and their clinical sequelae [1–3].

We adapted the 2018 ISHLT guidelines to grade individual airway anastomoses in a single-center cohort of lung transplant recipients to establish the incidence of clinically significant airway ischemia and identify the clinical and physiologic risk factors associated with this complication.

## Patients and Methods

### Patient Population

This was a retrospective, single-center study of all lung transplants performed between February 2016 and December 2020 at our institution. Patients were included if they had 6 months of bronchoscopic airway pictures available for grading and three-year clinical follow-up. Patients who died within this timeframe were included if postoperative airway images were available for review. Single, double, dual-organ and re-do lung transplants were included. This study was approved by the Baylor College of Medicine Institutional Review Board with waiver of consent.

### Surgical Technique

There were four surgeons who performed lung transplants during the study interval. Each surgeon utilized their preferred surgical technique for the airway anastomosis, including either an interrupted or running suture technique. Either of the following techniques were characterized as “interrupted suture technique”: 1) interrupted figure of 8 poly-p-diaxanone (PDS) for the cartilaginous portion and running PDS for the membranous portion or 2) interrupted 4-O prolene for the cartilaginous portion and running 4-O prolene for the membranous portion (our current center preference). The running suture technique was defined as the use of a single running circumferential suture line in a continuous fashion using 4-O prolene. All anastomoses were routinely reinforced with an onlay patch of donor pericardium. Patient medication and donor allograft preservation protocols are detailed in Supplementary Methods S1.

### Airway Grading

At our program, it is standard for the transplant pulmonologist to digitally archive two-dimensional color images of the anastomosis and distal airways. We reviewed both the images and the bronchoscopy reports for all patients who underwent lung transplant within the study interval. One of three transplant pulmonologists reviewed the images and reports obtained at time points closest to 15, 30, 60, 90, and 180 days after transplant. Airways were graded only if the pictures were available for review.

**FIGURE 1:**
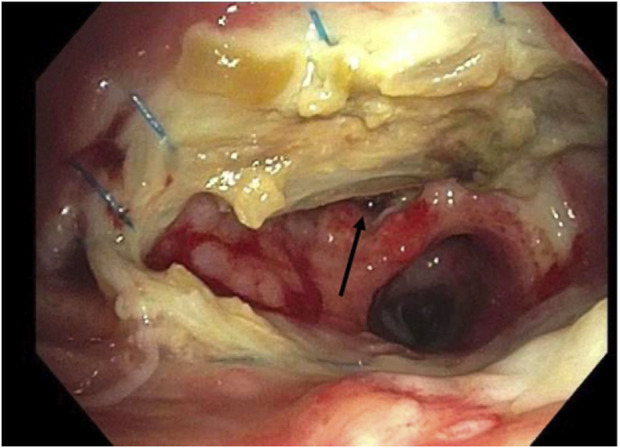
Example of grading airway ischemia. Right Anastomosis. Ischemia: C2. Stenosis: No. Dehiscence: Yes (arrow).

In 2018 the ISHLT convened a workgroup which proposed a detailed grading system for airway complications after transplant [15]. We modified this grading system to allow retrospective grading of archived images (Table 1). For example, ischemia and necrosis were combined into one category (“ischemia”) because they could not be easily distinguished on two-dimensional bronchoscopic digital images. We also simplified the reporting of dehiscence and stenosis for easier statistical analysis. Malacia was not evaluated as this diagnosis can only be confirmed by assessing the airway in motion. Figure 1 provides an example of how an anastomosis was graded in this study.

**TABLE 1:** Grading systems.

ISHLT’s proposed grading system		Our Study’s adapted grading system	
Ischemia andNecrosis (I)		Ischemia and Necrosis (I)	
*Location*	A. Perianastomotic - Within 1 cm of anastomosis	*Location*	A Perianastomotic - Within 1 cm of anastomosis
	B. Extending >1 cm from anastomosis to major airways (bronchus intermedium and distal left main-stem)		B. Extending >1 cm from anastomosis to major airways (bronchus intermedium and distal left main-stem)
	C. Extending >1 cm from anastomosis into lobar or segmental airways		C. Extending >1 cm from anastomosis into lobar and segmental airways
*Extent*	a. < 50% circumferential ischemia	*Extent*	1. < 50% circumferential ischemia or necrosis
	b. > 50%–100% circumferential ischemia		2. > 50%–100% circumferential ischemia or necrosis
	c. < 50% circumferential necrosis	**Dehiscence (D)**	Presence of any
	d. > 50%–100% circumferential necrosis	**Stenosis (S)**	
**Dehiscence (D)**		*Extent*	<50% stenosis
*Location*	a. Cartilaginous		>50% stenosis
	c. Membranous		
	c. Both		
*Extent*	a. 0%–25% of circumference		
	b. > 25%–50% of circumference		
	c. > 50%–75% of circumference		
	d. > 75% of circumference		
**Stenosis (S)**			
*Location*	a. Anastomotic		
	b. Anastomotic plus lobar/segmental		
	c. Lobar/segmental only		
Extent	a. 0%–25% reduction in cross-sectional area		
	b. > 25%–50% reduction in cross-sectional area		
	c. > 50% but <100% reduction in the cross-sectional area		
	d. 100% obstruction		
**Malacia (M)**			
Location	a. Perianastomotic - within 1 cm of anastomosis		
	b. Diffuse - involving anastomosis and extending beyond 1 cm		

### Clinically Significant Airway Ischemia

To facilitate reporting and analysis of bronchoscopic images, we simplified the reporting scheme to focus on a clinically significant and sensitive composite endpoint. We termed our composite endpoint “clinically significant airway ischemia” (CSAI), which was defined as the presence of airway ischemia severity >B2 (extending beyond 1 cm of the airway anastomosis and involving >50% of the anastomotic circumference), >50% stenosis, and/or presence of any dehiscence occurring at any timepoint within 6 months of the transplant. These findings were deemed clinically significant because they warranted either bronchoscopic interventions, changes in patient management or at least frequent bronchoscopies beyond routine surveillance procedures. Only balloon dilation and/or stent placement were considered interventions in this study.

### Study Outcomes

The primary outcome of this study was overall survival. The secondary outcomes included primary graft dysfunction (PGD), post-operative use of extracorporeal membrane oxygenation (ECMO), ventilation time, atrial fibrillation, major bleeding requiring take back to the operating room, acute cellular rejection, hospital and intensive care unit (ICU) length of stay (LOS), hospital readmission within 1 year, tracheostomy, acute kidney injury requiring dialysis, or pneumonia. PGD was defined as the presence of PGD grade 3 at 48 and/or 72 h post-reperfusion. Pneumonia was defined by positive bronchial cultures requiring antibiotic treatment. Finally, we sought to determine clinical and physiologic risk factors associated with CSAI using individual airways.

### Statistical Analysis

Continuous data are expressed as mean +/- standard deviation. Nominal variables are expressed as percentages. Statistical significance of continuous data was calculated using the unpaired two-tailed *t*-test for normally distributed variables and the Mann-Whitney *U*-test for variables showing a skewed distribution. Contingency analysis of nominal data was performed using a two-sided Fisher’s exact test. Both unadjusted and adjusted analysis were performed. For unadjusted analysis, a univariate logistic regression of CSAI of each factor was conducted, and the *p*-value of Student’s t-test, odds ratio, and 95% confidence interval were reported. A *p*-value of <0.05 was considered statistically significant. Contingency tables were made between CSAI and each categorical factor and computed for marginal percentage. For the adjusted analysis, a multivariate logistic regression model was employed, starting with a list of clinically significant variables identified *a priori* based on the published literature (Supplementary Table S1). This was followed by a forward stepwise variable selection process, guided by the Akaike information criterion (AIC), to selectively add factors from the unadjusted analysis that had a *p*-value <0.1. This approach was used to assess the strength of the association between CSAI and all potential risk factors and to determine the significant factors in the full model.

Actuarial survival rates were estimated with the Kaplan-Meier method and compared with the log-rank test. An adjusted analysis including recipient age, pulmonary artery pressures, PGD, and LAS was used to establish the association between CSAI and survival.

## Results

### Incidence and Outcomes Associated With Clinically Significant Airway Ischemia

**FIGURE 2:**
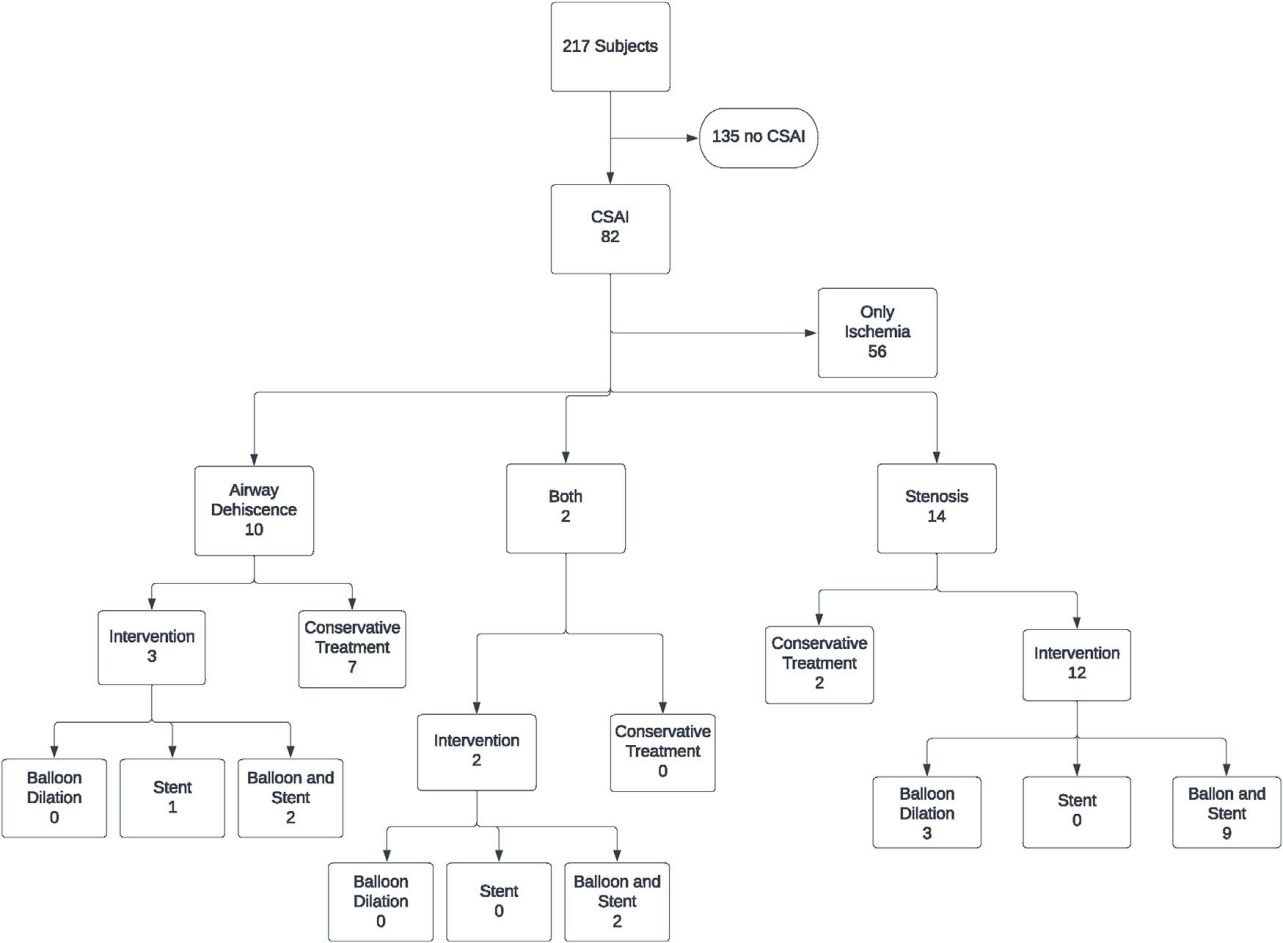
Breakdown of the patients who developed CSAI and associated interventions.

217 patients underwent lung transplantation between February 2016 and December 2020. Of these, 169 patients underwent double lung transplant, and 48 patients underwent single lung transplant. Bronchoscopic images were available for all 386 airway anastomoses. Eighty-two patients out of the 217 in the study cohort (37.8%) met the definition of CSAI in at least one of their airway anastomoses. Of these patients, 56 (68.3%) had ischemic lesions only and 26 (31.7%) had dehiscence and/or stenosis, with 17 (21%) requiring intervention (balloon dilation and/or stent placement) (Figure 2).

Supplementary Table S2 outlines the patient, donor, and operative characteristics between patients that did and did not develop CSAI. No statistically significant differences were identified between groups. Table 2 summarizes the postoperative outcomes associated with CSAI. One-year survival was lower in the CSAI group compared to the non-CSAI group (80.5% vs. 91.9%, *p* = 0.05). This reduction in one-year survival persisted after adjusting for recipient age, pulmonary artery pressure, PGD, and LAS. Three-year survival was non-significantly lower in the CSAI-group compared to the non-CSAI group (67.1% vs. 77.8%, *p* = 0.08). There was no difference in 90-day survival between the two groups. Additionally, we did not identify a difference in secondary outcome (Table 2).

**TABLE 2:** Patient outcomes.

Postoperative and survival outcomes with and without clinically significant airway ischemia			
	CSAI (N = 82)	Non-CSAI (N = 135)	*p*-values
**Postoperative Outcomes**			
PGD grade 3 at 48–72 h	24 (29.3%)	40 (29.6%)	0.95
Post-Op ECMO	11 (13.4%)	15 (11.1%)	0.61
Ventilator Support >5 days	24 (29.3%)	37 (27.4%)	0.77
Atrial Fibrillation	36 (43.9%)	60 (44.4%)	0.94
Major Bleeding	11 (13.4%)	10 (7.4%)	0.15
Acute Cellular Rejection	9 (11%)	8 (5.9%)	0.19
Hospital Length of Stay	32.68 (30.13)	25.90 (29.64)	0.12
ICU Length of Stay	20.77 (26.38)	15.43 (21.48)	0.11
Hospital Readmission within 1 year	66 (52.4%)	103 (39.6%)	0.09
Tracheostomy	21 (25.6%)	30 (22.2%)	0.57
Dialysis	12 (14.6%)	13 (9.6%)	0.27
Pneumonia	20 (24.4%)	23 (17.0%)	0.19
**Survival**			
90-day	78 (95.1%)	131 (97%)	0.47
1 year	66 (80.5%)	124 (91.9%)	0.05
3 years	55 (67.1%)	105 (77.8%)	0.08

Continuous variables expressed as Mean (SD); Categorical variables expressed as frequency (%).

CSAI: clinically significant airway ischemia, *PGD*: primary graft dysfunction.

**FIGURE 3:**
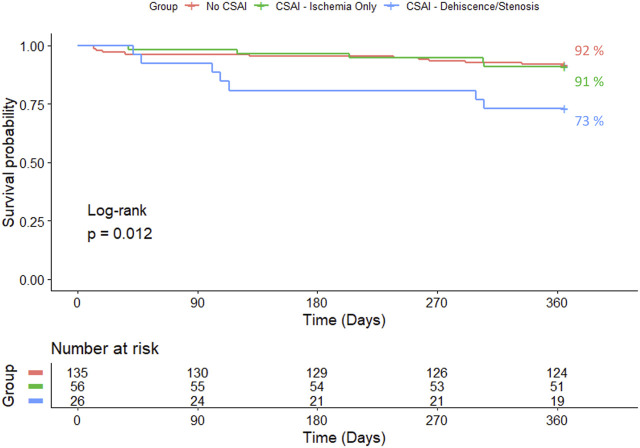
Kaplan-Meier 1-year survival estimates for patients without CSAI, with CSAI - Ischemia Only, and with CSAI—Dehiscence/Stenosis.

**FIGURE 4:**
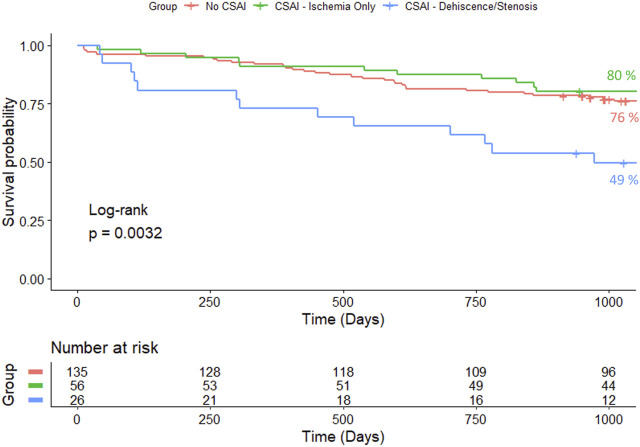
Kaplan-Meier 3-year survival estimates for patients without CSAI, with CSAI - Ischemia Only, and with CSAI—Dehiscence/Stenosis.

Figure 3 and Figure 4 show one and three-year survivals, respectively, for patients with no CSAI, CSAI patients with ischemia only, and CSAI patients with dehiscence and/or stenosis. The one-year survival rates were 92% for no CSAI, 91% for CSAI with ischemia only, and 73% for CSAI with dehiscence and/or stenosis (*p* = 0.012) (Figure 3). Three-year survival rates were 76% for no CSAI, 80% for CSAI with ischemia only, and 49% for CSAI with dehiscence and/or stenosis (*p* = 0.0032) (Figure 4). Thus, reduction in survival associated with CSAI appeared to be driven by the effect of dehiscence and/or stenosis. This was confirmed in a refined analysis using a Cox regression model, which showed there is a significant reduction in one-year survival in the CSAI with dehiscence and/or stenosis group compared with the non-CSAI group (*p* = 0.005) but no difference in one-year survival between the non-CSAI and CSAI with ischemia only groups (*p* = 0.46).

Overall, the average time between lung transplant and detection of CSAI was 23.5 days (SD + 14.2). In the subgroup of patients who only had CSAI with ischemia, the average time between lung transplant and detection of CSAI was 20.1 days (SD + 10). In the subgroup of 26 patients who had CSAI with dehiscence and/or stenosis, the average time between lung transplant to detection of airway ischemia was 32.3 days (SD + 19.1) and the average time from airway ischemia to detection of dehiscence and/or stenosis was 32.6 days (SD + 30.9). Every case of dehiscence and/or stenosis was preceded by bronchoscopic evidence of ischemia.

### Risk Factors for Clinically Significant Airway Ischemia

Of the 386 airway anastomoses reviewed, 222 (57.5%) were right-sided and 164 (42.5%) were left-sided. A total of 126 out of 386 (32.6%) anastomoses developed CSAI; 65 (51.6%) were right-sided and 61 (48.4%) were left-sided anastomoses.

Univariate analysis was performed to determine clinical and physiologic risk factors associated with airways that developed CSAI. Significant clinical risk factors included the following: lack of life support prior to lung transplant, recipient diabetes, intraoperative ECMO, use of a single running suture technique versus an interrupted suture technique, and major bleeding associated with takeback to the operating room (Table 3). The only significant physiologic risk factor associated with airways that developed CSAI in the unadjusted analysis was a reduced peak mixed venous oxygen saturation (MVO2) within 48 h (mean MVO2 of 72% in CSAI vs. 76% in no CSAI, *p* = .01) (Supplementary Table S3). Notably, lactate, vasopressor requirements, albumin, and hemoglobin values were not significantly associated with CSAI. Despite a trend towards greater donor culture positivity in the CSAI group, it did not reach significance.

**TABLE 3:** Unadjusted analysis.

Unadjusted analysis for risk factors associated with clinically significant airway ischemia					
	CSAI (N = 126)	Non-CSAI (N = 260)	*p*-value	OR	CI
**Patient Demographics**					
Age (years)	51.30 (15.86)	53.29 (15.31)	0.24	0.99	0.98, 1.01
Gender					
Female	52 (41.3%)	105 (40.4%)			
Male	74 (58.7%)	155 (59.6%)	0.87	0.96	0.63, 1.49
Body-Mass Index	25.36 (5.71)	25.39 (5.41)	0.96	1	0.96, 1.04
Type of Transplant					
Single	15 (11.9%)	33 (12.7%)			
Double	111 (88.1%)	227 (87.3%)	0.83	1.08	0.57, 2.11
First Anastomosis					
Left	61 (48.4%)	103 (39.6%)			
Right	65 (51.6%)	157 (60.4%)	0.1	0.7	0.45, 1.07
Primary Diagnosis					
ILD/Restrictive Lung Disease	68 (54%)	152 (58.5%)			
COPD	23 (18.3%)	42 (16.2%)	0.5	0.82	0.46, 1.48
Cystic Fibrosis	25 (19.8%)	47 (18.1%)	0.38	1.83	0.46, 7.21
PAH/PVD	5 (4%)	5 (1.9%)	0.94	0.97	0.48, 1.97
Other	5 (4%)	14 (5.4%)	0.46	0.65	0.19, 1.95
Multiorgan Transplant	4 (3.2%)	7 (2.7%)	0.79	1.19	0.31, 4.00
LAS score	43.10 (11.53)	45.79 (15.65)	0.09	0.99	0.97, 1.00
ABO Type					
A	46 (36.5%)	97 (37.3%)			
B	12 (9.5%)	26 (10%)	0.95	0.97	0.44, 2.07
O	61 (48.4%)	122 (46.9%)	0.82	1.05	0.66, 1.69
AB	7 (5.6%)	15 (5.8%)	0.97	0.98	0.36, 2.51
Condition at Transplant					
Hospitalized	4 (3.2%)	8 (3.1%)			
ICU	8 (6.3%)	31 (11.9%)	0.37	0.52	0.13, 2.32
Not hospitalized	114 (90.5%)	221 (85%)	0.96	1.03	0.32, 3.93
Life support prior to transplant	5 (4%)	28 (10.8%)	0.03	0.34	0.11, 0.84
Preoperative Ventilator Use	0 (0%)	4 (1.5%)	0.98	0	NA, 5.68E+29
Preoperative ECMO	2 (1.6%)	10 (3.8%)	0.22	0.38	0.06, 1.48
Preoperative Noninvasive Ventilation	3 (2.4%)	14 (5.4%)	0.17	0.41	0.09, 1.29
Mean PAP (mmHg)	26 (9.44)	26.80 (9.87)	0.71	1	0.97, 1.02
Creatinine (mg/dL)	0.86 (0.20)	0.86 (0.45)	0.93	0.97	0.50, 1.67
Prior cardiac surgery	0 (0%)	9 (3.5%)	0.98	0	NA, 1.23E+21
Prior lung surgery	23 (18.3%)	39 (15%)	0.42	1.27	0.71, 2.21
Type 2 diabetes mellitus	39 (31%)	55 (21.2%)	0.04	1.67	1.03, 2.70
History of Smoking	64 (50.8%)	134 (51.5%)	0.89	0.97	0.63, 1.49
Re-Transplant	1 (0.8%)	9 (3.5%)	0.16	0.22	0.01, 1.21
Chronic steroid use	46 (36.5%)	111 (42.7%)	0.25	0.77	0.50, 1.19
**Donor Characteristics**					
Donor Type					
DBD	120 (95.2%)	241 (92.7%)			
DCD	6 (4.8%)	19 (7.3%)	0.34	0.63	0.23, 1.54
Age (years)	36.16 (12.69)	35.07 (12.75)	0.43	1.01	0.99, 1.02
Gender					
Female	54 (42.9%)	86 (33.1%)			
Male	72 (57.1%)	174 (66.9%)	0.06	0.66	0.43, 1.02
Diabetes	16 (12.7%)	24 (9.2%)	0.3	1.42	0.72, 2.77
>20 py smoking history	78 (61.9%)	139 (53.5%)	0.13	1.4	0.91, 2.18
Extended Criteria Donor^a^	55 (43.7%)	102 (39.2%)	0.41	1.2	0.78, 1.85
Donor Cultures					
*Candida* species	41 (32.5%)	66 (25.4%)	0.10	1.49	0.93, 2.38
Any positive donor cultures	101 (80.2%)	202 (77.7%)	0.24	1.40	0.81, 2.51
**Perioperative Characteristics**					
ECLS					
Off-Pump	20 (15.9%)	63 (24.2%)			
ECMO	35 (27.8%)	57 (21.9%)	0.05	1.93	1.01, 3.77
CPB	71 (56.3%)	140 (53.8%)	0.11	1.6	0.91, 2.90
EVLP	28 (22.2%)	42 (16.2%)	0.15	1.48	0.86, 2.52
Total Ischemic Time (min)	330.00 (153.93)	320.95 (132.66)	0.55	1	1.00, 1.00
Warm Ischemic Time (min)	42.72 (14.84)	45.20 (13.00)	0.19	0.99	0.96, 1.01
Suture Technique					
Running	100 (79.4%)	171 (65.8%)			
Interrupted	26 (20.6%)	89 (34.2%)	0.01	0.5	0.30, 0.82
PGD 3 at 48–72 h	28 (22.2%)	57 (21.9%)	0.95	1.02	0.60, 1.69
Post-Op ECMO	18 (14.3%)	33 (12.7%)	0.67	1.15	0.61, 2.11
Ventilator Support >5 days	40 (31.7%)	76 (29.2%)	0.61	1.13	0.71, 1.78
Peak mixed venous O2 within 48 h	72.46 (11.55)	76.00 (10.02)	0.01	0.97	0.95, 0.99
Peak Creatinine within 48 h	1.02 (0.34)	1.04 (0.50)	0.61	0.88	0.53, 1.41
Peak Lactate within 72 h	7.10 (3.62)	6.96 (3.72)	0.73	1.01	0.95, 1.07
Atrial Fibrillation	56 (44.4%)	110 (42.3%)	0.69	1.09	0.71, 1.67
Major Bleeding^b^	21 (16.7%)	19 (7.3%)	0.01	2.54	1.31, 4.95
Acute Cellular Rejection	14 (11.1%)	16 (6.2%)	0.09	1.9	0.88, 4.03

Continuous variables expressed as Mean (SD); Categorical variables expressed as frequency (%).

^a^ Extended Criteria Donor: Age >55, DCD, PF < 300, Anticipated ischemia >6 h, abnormal chest X-ray, >20 py smoking history.

^b^ Major bleeding within the early postoperative period requiring surgical intervention.

CSAI: clinically significant airway ischemia, ILD: interstitial lung disease, PAH: pulmonary arterial hypertension, PVD: pulmonary vascular disease, LAS: lung allocation score, ICU: intensive care unit, ECMO: Extra-Corporeal Membrane Oxygenation, PAP: pulmonary arterial pressure, DBD: donor after brain death, DCD: donor after circulatory death, CPB: Cardio-Pulmonary Bypass, EVLP: *Ex-Vivo* Lung Perfusion.

PGD: primary graft dysfunction.

The adjusted analysis identified the following risk factors for CSAI: younger recipient age, diabetes in the recipient, performance of the left anastomosis first, single running suture technique versus an interrupted suture technique, and major bleeding associated with takeback to the operating room (Table 4). Of note, not all patients had a MVO2 drawn after transplant. Thus, a similar adjusted analysis using stepwise variable selection with AIC was performed with a smaller cohort of airways (n = 306) from patients that had complete MVO2 data. This analysis showed that a higher peak MVO2 within 48 h after transplant was associated with a reduced risk of CSAI (Supplementary Table S4).

**TABLE 4:** Adjusted analysis.

Adjusted analysis for risk factors associated with clinically significant airway ischemia			
	*p*-value	OR	CI
Recipient age (years)	0.09	0.98	0.95, 1.00
Recipient gender: Male (vs. Female)	0.16	1.51	0.86, 2.72
Type of Transplant: Single (vs. Double)	0.11	0.51	0.22, 1.17
First Anastomosis: Right (vs. Left)	**0.01**	0.54	0.32, 0.88
Primary Diagnosis			
ILD/Restrictive Lung Disease			
COPD	0.19	1.57	0.79, 3.08
Cystic Fibrosis	0.32	0.57	0.19, 1.72
PAH/PVD	0.68	1.37	0.29, 6.27
Other	0.27	0.37	0.05, 1.76
Condition at Transplant			
Not hospitalized			
ICU	0.99	1.01	0.29, 3.29
Hospitalized	0.39	0.46	0.06, 2.28
Life support prior to transplant	0.06	0.24	0.05, 1.00
Type 2 diabetes mellitus	0.06	1.79	0.97, 3.29
*Candida* albicans	0.12	1.61	0.88, 2.92
Donor gender: Male (vs. Female)	0.08	1.69	0.94, 3.08
Total Ischemic Time (min)	0.71	1.00	1.00, 1.00
Suture Technique: Interrupted (vs. Running)	**0.01**	0.47	0.25, 0.84
PGD 3 at 48–72 h	0.76	0.90	0.44, 1.78
Ventilator Support >5 days	0.77	1.10	0.58, 2.05
Major Bleeding^a^	0.10	1.89	0.88, 4.08
Acute Cellular Rejection	0.20	1.80	0.73, 4.43
Pneumonia	0.18	1.49	0.83, 2.64

^a^ Major bleeding within the early postoperative period, requiring surgical intervention. Bold values represent clinically significant *p*-values.

## Discussion

Our study incorporated the 2018 ISHLT consensus-based guidelines to retrospectively grade airway anastomoses in our center and to identify the incidence, risk factors, and outcomes associated with clinically significant airway complications. We found the grading system to be practical, reproducible, and efficient with only minor modifications needed for retrospective analysis of bronchoscopic images. We focused on a clinically significant composite outcome of the grading system which was the presence of any of the following: >B2 severity ischemia, >50% stenosis, and/or any evidence of dehiscence occurring at any time within 6 months of transplant.

The incidence of CSAI in our cohort was 37.8%. This was at the higher end of the 1.4%–38% range reported in recent series on post-transplant airway complications [1–3, 8, 11, 16]. Our higher incidence of airway complications was likely due to the sensitivity of our composite outcome which included ischemic lesions (>B2) with or without bronchoscopic interventions, many of which may not have qualified as an airway complication in other studies. However, we believe that these precursor lesions are important as evidenced by the high rate of dehiscence and stenosis (32%) seen in patients that developed > B2 ischemia. The incidence of dehiscence and/or stenosis in our study cohort was 11.9%, and the incidence of airway complications requiring interventions was 7.8%. These rates are similar to those reported in the literature [11, 13, 17, 18]. Also, like prior reports, our study showed that patients that developed CSAI with dehiscence and/or stenosis were at significantly greater risk of having reduced survival than patients that developed CSAI with ischemia only [1, 2].

We identified several risk factors associated with CSAI on multivariate analysis. Airway anastomosis with interrupted sutures along the anterior cartilaginous portion of the airway and a running posterior membranous suture line was superior to a single running Prolene suture. Of note, we routinely trim back the airway as close as possible to the secondary carina as suggested by several authors [17, 18, 20–22]. This modification to reduce the length of the bronchus has been key for reducing airway ischemia over the past two decades. The finding of an association between interrupted suture technique and reduction in airway ischemic complications has been observed by others [18, 20].

However, this finding is not ubiquitous. Schweiger et al. reported a low rate of severe airway complications requiring interventions in their series of lung transplants using exclusively a single running technique [23]. In contrast, their study did not have a comparison group, did not focus on early ischemic lesions, and did not have all bronchoscopic images available for review. Olland et al. also showed that a single running suture technique was not associated with increased airway complications if the donor airway was trimmed back substantially to include a wedge of the bronchus intermedius [24]. This modification was first described by Weder et al. who showed that extensive donor bronchial trimming on the left and the right was associated with a near absence of airway stenosis [25]. Unlike the study by Olland, Weder utilized an interrupted suture technique in their series. We hypothesize that interrupted sutures provide two advantages: 1) greater opportunity for microvascular connections and oxygen delivery, and 2) better alignment of the airway anastomosis.

Our multivariate analysis suggested that diabetes in a recipient was associated with greater odds of CSAI. This is consistent with the study by Olland et al., which found that recipient diabetes was independently associated with airway complications after transplant [24]. Diabetes affects the microvascular beds increasing the risk of tissue ischemia. Whether postoperative control of hyperglycemia is associated with reduced CSAI is intriguing and requires further investigation.

Major bleeding requiring takeback to the operating room was also a risk factor that, to our knowledge, has not been previously reported. We hypothesize that acute bleeding results in hypotension and prioritization of blood distribution to vascular beds in critical need leaving the airway anastomosis more vulnerable to ischemia. The association between major bleeding and CSAI underscores the potential vulnerability of the anastomosis to systemic changes that affect oxygen delivery and the healing milieu. However, it is important to note that neither nadir hemoglobin levels, nor vasopressor requirements were associated with CSAI. Perhaps the airway anastomosis is only vulnerable to major changes in these values associated with a takeback for bleed. Transient fluctuations in hemodynamics and blood requirements during the takeback for bleeding were not captured in this study.

Our multivariate analysis suggests that a right lung-first approach is associated with less risk of CSAI than a left lung-first approach. This finding requires further analysis, and we would not advocate for one approach over the other based on this finding alone. It is possible, however, that the airway anastomosis is subject to different perfusion patterns depending on which lung is implanted first. For example, when the right lung is implanted first, there is more space for it to ventilate and perfuse while working on the left lung. Conversely, when the left lung is implanted first, there may be less space to ventilate and perfuse because of external compression from the heart.

Importantly, we found that the peak MVO2 level was inversely associated with CSAI. Therefore, patients with a greater oxygen content in the pulmonary artery circulation had a lower rate of severe ischemia. This is also intuitive because, in the absence of bronchial artery reconstruction, the pulmonary artery is the sole blood supply to the transplanted lung. Maneuvers to increase the amount of oxygen in the venous return may be advantageous for reducing the risk of airway ischemia although this requires further study.

There were a few findings that were counterintuitive. The association between older age and reduced risk of airway ischemia was difficult to explain. The ages between patients in the CSAI and non-CSAI groups were similar. It was only after incorporating age as a previously reported risk factor, that we obtained a significant odds ratio suggesting an inverse relationship between age and airway risk. This finding requires further study. One possible explanation is that older patients free of comorbid conditions such as diabetes are more likely to receive lung transplant than those with multiple comorbid conditions. At our program, older recipients are more likely to receive single lung transplants to reduce surgical stress. In addition, postoperative albumin levels were not associated with CSAI. This is counterintuitive because one would assume that a lower albumin level would suggest worse nourishment and diminished wound healing. Perhaps this is explained by the low number of recipients in our cohort that were malnourished during the preoperative period. Our program makes every effort to optimize patient nutrition and weight prior to transplant.

In our series, severe airway ischemia was first detected approximately 4 weeks after transplant. Ischemic airways that went on to develop dehiscence and/or stenosis did so, on average, 4 weeks after the detection of ischemia. All airways that developed dehiscence/stenosis had evidence of severe ischemia first (i.e., > B2 by 2018 ISHLT guidelines - defined as ischemia >1 cm from anastomosis and >50% of the circumference). Thus, ischemia of this severity is an important precursor for greater complications. Frequent monitoring for progression or resolution of ischemia may improve outcomes through prompt recognition and treatment of advanced lesions [2, 3, 13, 26].

Prior studies have utilized novel grading systems for airway complications after transplantation. Yserbyt et al. utilized the MDS classification system in 2016 and performed a similar analysis looking at severe and less severe airway grades [21]. Contrary to our results, they showed that advanced recipient age was associated with airway complications and that right-sided anastomoses were at greater risk of complications than left sided anastomosis. We found that older age recipients were at lower risk of airway complications, and we did not find a difference in laterality although we noticed a trend towards greater complications in the right-sided anastomoses. The difference in results could certainly be due to differences in patient cohorts as well as the differences in airway grading schemes. Yserbyt et al also determined that recipient microbiological colonization and postoperative infections were associated with airway complications. Olland et al. also identified postoperative infections as being important for the development of airway complications [24]. In our study patients with CSAI did not have higher rates of post-operative pneumonia compared to those without CSAI. Additionally, while we noted a trend towards greater donor culture positivity and incidence of *candida* species in patients with CSAI compared to no CSAI, these differences were not statistically significant in our cohort. It is known that fungal infections are a significant risk factor for airway complications [27]; however, our use of fungal prophylaxis with voriconazole or itraconazole has likely reduced this risk. It is conceivable that these trends could have been significant if we had analyzed a greater number of patients.

Moreover, previous literature has shown associations between various additional risk factors and airway complications. A retrospective study of the United Network for Organ Sharing (UNOS) database evaluated risk factors associated with airway complications [1]. They showed an incidence of 1.4% and found the following risk factors: ICU hospitalization before transplant, advanced recipient age, male recipient, bilateral lung transplantation, and diagnosis other than emphysema, cystic fibrosis, or idiopathic pulmonary fibrosis. We did not identify these risk factors, although this could be due to differences in the airway grading, transplant eras, patient population, and inclusion of covariates.

Other series have reported unique risk factors such as: donor and recipient ventilation times, early rejection, donor recipient size mismatch, cold ischemic interval, and PGD [1, 2, 8, 9, 16–19, 24, 28]. We did not study donor ventilation times in the current analysis. However, we were surprised that total organ ischemic time, PGD, and recipient ventilation times were not associated with airway complications in our series. It is conceivable that with a larger sample size, these factors may emerge as significantly associated with CSAI and at present we would not dismiss them as being potentially important factors affecting airway healing.

Our study has several limitations. Its retrospective nature relies on accurate chart review and assessment of airways. Grading airways remains somewhat subjective and biased, and grading retrospectively from 2-D bronchoscopic images is less reliable than grading them in real time. To mitigate this, prior to the study, pulmonologists graded a sample of airways to ensure consistency. This study modified the ISHLT 2018 grading system by combining ischemia and necrosis into one category as it is difficult to distinguish between the two from retrospective review of images. Our team recognizes that combining ischemia and necrosis could have led to an overestimation of the incidence of airway complications in our study group. On the other hand, our study suggests that without dehiscence/stenosis, isolated ischemic lesions had little impact on survival. Our study did not look at malacia because this diagnosis requires bronchoscopic visualization on forced exhalation, and it is a complication that may not be seen within 180 days [4]. As mentioned previously, we included presence of any dehiscence or stenosis rather than specifying the exact location of these lesions as suggested by the ISHLT 2018 airway guidelines. We agree that real time imaging and reporting of exact locations is ideal, however this was not possible in our current analysis. We also recognize that modifying the ISHLT grading system undermines its purpose of standardization and that the scoring guidelines were not intended to prognosticate patient outcomes. Despite the study’s limitations, it provides one of the largest series with 386 graded airways across multiple time-points. This is an important contribution to our existing knowledge of airway complications after lung transplant.

In conclusion, CSAI was a common complication after lung transplantation in our large single center experience. This complication was associated with reduced patient survival. However, this reduction in patient survival was driven by dehiscence/stenosis rather than by severe ischemia alone. While ischemia alone was not associated with reduced survival, it was an important precursor to severe complications. The proposed 2018 ISHLT guidelines for grading airway complications are functional in clinical practice and useful for standardizing the reporting of important post-transplant airway complications. Our findings establish the utility of the updated guidelines while highlighting potential methods to mitigate the risk of airway ischemia: achievement of euglycemia in diabetic recipients, establishment of hemostasis and avoidance of take back for bleeds, optimization of MVO2 levels, and use of interrupted suture technique for the airway anastomosis. Prospective research should evaluate these findings using real time bronchoscopic images across multiple centers.

## Data Availability

The original contributions presented in the study are included in the article/Supplementary Material, further inquiries can be directed to the corresponding author.

## Abbreviations

AIC, Akaike information criterion; CSAI, Clinically significant airway ischemia; ECD, Extended criteria donors; ECMO, Extracorporeal membrane oxygenation; EVLP, *Ex-vivo* lung perfusion; ICU, Intensive care unit; ISHLT, International Society of Heart and Lung Transplantation; LAS, Lung allocation score; LOS, Length of stay; MDS, Macroscopic aspect, diameter of anastomosis, sutures of anastomosis; MVO2, Mixed Venous Oxygen Saturation; OCS, Organ care system; PGD, Primary graft dysfunction; SRTR, Scientific Registry for Transplant Research; TEGLA, Thickness, extent of injury, granulation tissue, loose sutures, anastomotic complications; UNOS, United Network for Organ Sharing.
