# Supplementary material for: Risk Factors, Incidence, and Outcomes Associated With Clinically Significant Airway Ischemia
Source: Transpl Int. 2024 May 10;37:12751. doi: 10.3389/ti.2024.12751 (PMC11119282; doi:10.3389/ti.2024.12751)
Supplement: Supplementary file 1 [file Table1.docx]

**Variables associated with airway ischemia**

| **Variable** | **Reference** |
| --- | --- |
| Recipient Age | Santacruz, 2009^13^; Hayanga, 2016^1^; Murthy, 2007^2^; Yserbyt, 2016^23^ |
| Recipient Gender | Santacruz, 2009^13^; Hayanga, 2016^1^; Van De Wauwer, 2007^17^; Fitzsullivan, 2011^18^ |
| Type of Transplant | Santacruz, 2009^13^; Hayanga, 2016^1^; |
| Primary Diagnosis | Hayanga, 2016^1^; Murthy, 2007^2^ |
| Condition at Transplant | Hayanga, 2016^1^ |
| Life support prior to Transplant | Hayanga, 2016^1^; Crespo, 2018^15^ |
| Type 2 Diabetes Mellitus | Hayanga, 2016^1^ |
| Total ischemic time | Santacruz, 2009^13^; Crespo, 2021^11^; Crespo, 2018^15^ |
| Suture Technique | Santacruz, 2009^13^; Mahajan, 2017^4^; Crespo, 2021^11^; Murthy, 2007^2^; Crespo, 2018^15^; Van De Wauwer, 2007^17^; Fitzsullivan, 2011^18^ |
| PGD 3 at 48-72 hrs. | Santacruz, 2009^13^; Mahajan, 2017^4^; Crespo, 2021^11^; Crespo, 2018^15^ |
| Prolonged ventilator support | Santacruz, 2009^13^; Crespo, 2021^11^; Crespo, 2018^15^; Fitzsullivan, 2011^18^ |
| Acute Cellular Rejection | Santacruz, 2009^13^; Mahajan, 2017^4^; Crespo, 2018^15^; Fitzsullivan, 2011^18^ |
