# Supplementary material for: Risk Factors, Incidence, and Outcomes Associated With Clinically Significant Airway Ischemia
Source: Transpl Int. 2024 May 10;37:12751. doi: 10.3389/ti.2024.12751 (PMC11119282; doi:10.3389/ti.2024.12751)
Supplement: Supplementary file 2 [file Table2.docx]

| **Supplementary Table 2. Recipient and Donor Demographics and Operative Characteristics** | | | |
| --- | --- | --- | --- |
| **Recipient and donor demographics and operative characteristics with and without clinically significant airway ischemia** | | | |
|  | **CSAI (N=82)** | **Non-CSAI (N=135)** | **P-value** |
| **Patient Demographics** |  |  |  |
| Age (years) | 52.22 (16.21) | 55.39 (14.77) | 0.14 |
| Gender |  |  |  |
| Female | 32 (39%) | 54 (40%) |  |
| Male | 50 (61%) | 81 (60%) | 0.89 |
| Body-Mass Index | 25.39 (5.54) | 25.66 (5.35) | 0.72 |
| Type of Transplant |  |  |  |
| Single | 15 (18.3%) | 33 (24.4%) |  |
| Double | 67 (81.7%) | 102 (75.6%) | 0.29 |
| First Anastomosis |  |  |  |
| Left | 39 (47.6%) | 53 (39.3%) |  |
| Right | 43 (52.4%) | 82 (60.7%) | 0.23 |
| Primary Diagnosis |  |  |  |
| ILD / Restrictive Lung Disease | 44 (53.7%) | 85 (63%) |  |
| COPD | 16 (19.5%) | 21 (15.6%) | 0.31 |
| Cystic Fibrosis | 16 (19.5%) | 20 (14.8%) | 0.49 |
| PAH / PVD | 3 (3.7%) | 2 (1.5%) | 0.92 |
| Other | 3 (3.7%) | 7 (5.2%) | 0.45 |
| Multiorgan Transplant | 2 (2.4%) | 4 (3%) | 0.82 |
| LAS score | 43.38 (12.20) | 45.16 (15.10) | 0.37 |
| ABO Type |  |  |  |
| A | 30 (36.6%) | 51 (37.8%) |  |
| B | 9 (11%) | 14 (10.4%) | 0.85 |
| O | 39 (47.6%) | 62 (45.9%) | 0.83 |
| AB | 4 (4.9%) | 8 (5.9%) | 0.80 |
| Condition at Transplant |  |  |  |
| Hospitalized | 2 (2.4%) | 4 (3%) |  |
| ICU | 6 (7.3%) | 14 (10.4%) | 0.88 |
| Not hospitalized | 74 (90.2%) | 117 (86.7%) | 0.79 |
| Life support prior to transplant | 5 (6.1%) | 12 (8.9%) | 0.46 |
| Preoperative Ventilator Use | 0 (0%) | 2 (1.5%) | 0.99 |
| Preoperative ECMO | 2 (2.4%) | 4 (3%) | 0.80 |
| Preoperative Noninvasive Ventilation | 3 (3.7%) | 6 (4.4%) | 0.76 |
| Mean PAP (mmHg) | 26.12 (9.66) | 26 (9.46) | 0.93 |
| Creatinine (mg/dL) | 0.86 (0.19) | 0.87 (0.45) | 0.87 |
| Prior cardiac surgery | 0 (0%) | 6 (4.4%) | 0.99 |
| Prior lung surgery | 14 (17.1%) | 22 (16.3%) | 0.88 |
| Type 2 diabetes mellitus | 24 (29.3%) | 28 (20.7%) | 0.16 |
| History of Smoking | 45 (54.9%) | 72 (53.3%) | 0.82 |
| Re-Transplant | 1 (1.2%) | 5 (3.7%) | 0.30 |
| Immunosuppression | 29 (35.4%) | 63 (46.7%) | 0.10 |
| Chronic steroid use | 30 (36.6%) | 58 (43%) | 0.35 |
|  |  |  |  |
| **Donor Demographics** |  |  |  |
| Donor |  |  |  |
| DBD | 77 (93.9%) | 127 (94.1%) |  |
| DCD | 5 (6.1%) | 8 (5.9%) | 0.96 |
| Age (years) | 36.71 (12.92) | 34.50 (12.66) | 0.22 |
| Gender |  |  |  |
| Female | 33 (40.2%) | 42 (31.1%) |  |
| Male | 49 (59.8%) | 93 (68.9%) | 0.06 |
| Diabetes | 16 (19.5%) | 24 (17.8%) | 0.17 |
| > 20 py smoking history | 50 (61%) | 71 (52.6%) | 0.25 |
| Extended Criteria Donor | 38 (46.3%) | 50 (37%) | 0.18 |
|  |  |  |  |
| **Operative Characteristics** |  |  |  |
| ECLS |  |  |  |
| Off-Pump | 17 (20.7%) | 43 (31.9%) |  |
| ECMO | 21 (25.6%) | 26 (19.3%) | 0.08 |
| CPB | 44 (53.7%) | 66 (48.9%) | 0.13 |
| EVLP | 19 (23.2%) | 19 (14.1%) | 0.09 |
| Total Ischemic Time (min) | 368.57 (162.41) | 348.10 (124.90) | 0.30 |
| Continuous variables expressed as Mean (SD); Categorical variables expressed as frequency (%). *CSAI:*  Clinically Significant Airway Ischemia, *ILD:* Interstitial Lung Disease, *PAH:* Pulmonary Arterial Hypertension, *PVD*: Pulmonary Vascular Disease, *LAS*: Lung Allocation Score, *ICU*: Intensive Care Unit, *ECMO*: Extra-Corporeal Membrane Oxygenation, *PAP*: Pulmonary Arterial Pressure, *DBD*: Donor after Brain Death, *DCD*: Donor after Circulatory Death, *CPB:* Cardio-Pulmonary Bypass, *EVLP*: Ex-Vivo Lung Perfusion. | | | |
