# Supplementary material for: Risk Factors, Incidence, and Outcomes Associated With Clinically Significant Airway Ischemia
Source: Transpl Int. 2024 May 10;37:12751. doi: 10.3389/ti.2024.12751 (PMC11119282; doi:10.3389/ti.2024.12751)
Supplement: Supplementary file 3 [file Table3.docx]

| **Supplementary Table 3. Physiological Risk Factors** | | | | | |
| --- | --- | --- | --- | --- | --- |
| Laboratory biomarkers associated with clinically significant airway ischemia | | | | | |
|  | **CSAI**  **(N=126)** | **Non-CSAI**  **(N=260)** | **P-value** | **OR** | **CI** |
| Peak lactate within 72 hrs. | 7.10 (3.62) | 6.96 (3.72) | 0.73 | 1.01 | 0.95, 1.07 |
| Vasopressors* |  |  |  |  |  |
| Epinephrine | 54 (42.9%) | 125 (48.1%) | 0.27 | 0.78 | 0.50, 1.21 |
| Norepinephrine | 83 65.9%) | 155 (59.6%) | 0.33 | 1.28 | 0.78, 2.10 |
| Vasopressin | 73 (57.9%) | 136 (52.3%) | 0.53 | 1.17 | 0.73, 1.88 |
| Phenylephrine | 2 (1.6%) | 2 (0.8%) | 0.52 | 1.93 | 0.23, 16.52 |
| Angiotensin II | 1 (0.8%) | 2 (0.8%) | 0.99 | 1.01 | 0.05, 10.88 |
| Peak mixed venous O2^+^ within 48 hrs. | 72.46 (11.55) | 76.00 (10.02) | **0.01** | **0.97** | **0.95, 0.99** |
| Nadir mixed venous O2^+^ within 48 hrs. | 63.83 (12.64) | 64.76 (11.45) | 0.52 | 0.99 | 0.97, 1.01 |
| Mean mixed venous O2^+^within 48 hrs. | 68.7 (10.26) | 70.08 (9.07) | 0.23 | 0.98 | 0.96, 1.01 |
| First postoperative albumin | 3.16 (0.47) | 3.17 (0.57) | 0.81 | 0.95 | 0.64, 1.42 |
| Nadir hemoglobin within 48 hrs. | 8.28 (1.35) | 8.42 (1.47) | 0.38 | 0.94 | 0.80, 1.09 |
| Peak Total Bilirubin within 72 hrs. | 2.17 (1.44) | 2.14 (1.25) | 0.83 | 1.02 | 0.86, 1.19 |
| Peak Creatinine within 48 hrs. | 1.02 (0.34) | 1.04 (0.50) | 0.61 | 0.88 | 0.53, 1.41 |
| * Vasopressors utilized within 4 hours after reperfusion.  ^+^Mixed venous O2 obtained from a central venous line | | | | | |
