# Supplementary material for: Risk Factors, Incidence, and Outcomes Associated With Clinically Significant Airway Ischemia
Source: Transpl Int. 2024 May 10;37:12751. doi: 10.3389/ti.2024.12751 (PMC11119282; doi:10.3389/ti.2024.12751)
Supplement: Supplementary file 4 [file Table4.docx]

| **Supplementary Table 4. Subgroup Adjusted Analysis** | | | |
| --- | --- | --- | --- |
| Adjusted analysis for risk factors associated with clinically significant airway ischemia. | | | |
|  | **P-value** | **OR** | **CI** |
| Recipient age (years) | 0.14 | 0.98 | 0.95, 1.01 |
| Recipient gender: Male (vs. Female) | **<0.01** | 3.16 | 1.46, 6.82 |
| Type of Transplant: Single (vs. Double) | **0.04** | 0.37 | 0.15, 0.94 |
| Primary Diagnosis |  |  |  |
| ILD / Restrictive Lung Disease |  |  |  |
| COPD | 0.54 | 1.27 | 0.59, 2.77 |
| Cystic Fibrosis | 0.86 | 1.12 | 0.31, 4.04 |
| PAH / PVD | 0.17 | 3.74 | 0.58, 24.29 |
| Other | 0.98 | 0.00 | NA, NA |
| Condition at Transplant |  |  |  |
| Not hospitalized |  |  |  |
| ICU | 0.94 | 1.04 | 0.31, 3.55 |
| Hospitalized | 1.00 | 0.11 | NA, NA |
| Life support prior to transplant | 0.08 | 0.23 | 0.05, 1.17 |
| Type 2 diabetes mellitus | **0.05** | 1.98 | 0.99, 3.97 |
| Candida albicans | 0.18 | 1.62 | 0.80, 3.29 |
| Donor gender: Male (vs. Female) | **0.01** | 2.53 | 1.23, 5.21 |
| EVLP | 0.12 | 2.51 | 0.79, 7.98 |
| Total Ischemic Time (min) | 0.65 | 1.00 | 1.00, 1.00 |
| Suture Technique: Interrupted (vs. Running) | 0.06 | 0.54 | 0.28, 1.03 |
| PGD 3 at 48-72 hrs. | 0.60 | 1.25 | 0.55, 2.76 |
| Ventilator Support > 5 days | 0.62 | 1.20 | 0.59, 2.43 |
| Major Bleeding* | **0.03** | 2.56 | 1.07, 6.14 |
| Acute Cellular Rejection | 0.89 | 1.08 | 0.38, 3.05 |
| Pneumonia | 0.47 | 1.29 | 0.65, 2.55 |
| Peak mixed venous O2 | **<0.01** | 0.96 | 0.94, 0.99 |
| * Major bleeding within the early postoperative period, requiring surgical intervention. | | | |
