# Supplementary material for: Risk Factors, Incidence, and Outcomes Associated With Clinically Significant Airway Ischemia
Source: Transpl Int. 2024 May 10;37:12751. doi: 10.3389/ti.2024.12751 (PMC11119282; doi:10.3389/ti.2024.12751)
Supplement: Supplementary file 5 [file DataSheet1.docx]

**Medications**

Per institution protocol, patients underwent standard induction treatment with basiliximab, mycophenolate mofetil, and high-dose methylprednisolone taper followed by three drug (tacrolimus, mycophenolate mofetil and prednisone) immunosuppression as tolerated. Perioperative empiric antibiotics included vancomycin and cefepime, which were adjusted based on donor and surgical cultures obtained at the time of transplant. Prophylaxis antibiotics included valganciclovir, itraconazole or voriconazole, and sulfamethoxazole - trimethoprim.

**Donor preservation**

For standard ice donor lung preservation, lungs were flushed antegrade with 4 liters cold Perfadex solution and retrograde with 2 liters cold Perfadex solution. Lungs were packaged and transported in an ice cooler. Portable ex-vivo lung perfusion (EVLP) with Organ Care System (OCS) Lung was used primarily for extended criteria donors (ECD) which included any one or a combination of the following donor features: age > 55 years, > 20 pack-year smoking history, > 6-hr anticipated ischemia time, donation after circulatory arrest, or PaO2:FiO2 ratio <300^1^. Standard OCS procurement and preservation protocols were followed in these cases^2-5^.

**Airway grading**

Each airway was graded by one of three transplant pulmonologists, and the grading system for the study was adopted from the ISHLT guidelines (Table 1). It is standard practice at our center for the pulmonologist to obtain and save pictures of each anastomosis at the time of bronchoscopy. Bronchoscopy reports for the first 6 months after transplant were reviewed for each patient, and airways were graded from bronchoscopy reports closest to 15, 30, 60, 90, and 180 days after transplant.  Airways were graded only if the pictures were available to review. Figure 1 exemplifies how we graded an anastomosis. We did not include malacia as it is typically diagnosed real time on bronchoscope exam.  Also, we combined ischemia and necrosis because it was difficult to distinguish between the two on bronchoscopic pictures.

**Physiologic Risk Factors**

Physiologic risk factors included laboratory biomarkers of end organ function shortly after the lung transplant such as peak lactate levels, mixed venous oxygen saturation, nadir hemoglobin, total bilirubin, and others. We also included pressor requirements as a physiologic risk factor. Mixed venous oxygen saturation was collected via a central venous catheter.

**References**

1. van Berkel V, Guthrie TJ, Puri V, et al. Impact of anastomotic techniques on airway complications after lung transplant. *Ann Thorac Surg.* 2011;92(1):316-320; discussion 320-311.
2. Warnecke G, Van Raemdonck D, Smith M, et al. (242) - The Organ Care System (OCS™) Lung INSPIRE International Trial Results. *The Journal of Heart and Lung Transplantation.* 2015;34(4, Supplement):S96.
3. Loor G, Warnecke G, Villavicencio MA, et al. Portable normothermic ex-vivo lung perfusion, ventilation, and functional assessment with the Organ Care System on donor lung use for transplantation from extended-criteria donors (EXPAND): a single-arm, pivotal trial. *The lancet respiratory medicine.* 2019;7(11):975-984.
4. Warnecke G, Van Raemdonck D, Smith MA, et al. Normothermic ex-vivo preservation with the portable Organ Care System Lung device for bilateral lung transplantation (INSPIRE): a randomised, open-label, non-inferiority, phase 3 study. *The lancet respiratory medicine.* 2018;6(5):357-367.
5. Loor G. EVLP: Ready for Prime Time? *Seminars in thoracic and cardiovascular surgery.* 2019;31(1):1-6.
